# Do we need to demonstrate amyloid in tissue for hereditary ATTR amyloidosis ?

**DOI:** 10.1186/1750-1172-10-S1-I25

**Published:** 2015-11-02

**Authors:** Per Westermark

**Affiliations:** 1 Department of Immunology, Genetics and Pathology, Uppsala University, Uppsala, Sweden

## 

Amyloidosis is by definition deposition of aggregates of proteins in a characteristic β-pleated sheet fibrillar conformation. Amyloid is recognized by its histological appearance particularly after some specific stainings among which Congo red most commonly is used. A green birefringence is the hallmark of all kinds of amyloid. There is a number of emerging diagnostic techniques based on labelling with antibodies, other proteins and some other ligands that are at least to some degree specific for amyloid and may be used for *in vivo* detection of amyloid. With some exceptions, such techniques are still not sensitive enough to detect small deposits. Biopsy stained with Congo red and examined in a polarization microscope is necessary presently.

Hereditary ATTR amyloidosis constitutes a heterogeneous disease group with varying organ consequences, not only from peripheral nerves and heart. Amyloid staining properties vary and Congo red affinity and birefringence may be very weak, particularly in amyloid consisting of TTR fragments which is common (1). Therefore is amyloid sometimes difficult to recognize also in biopsies.

Given the varying clinical manifestation of ATTR amyloidosis it is my opinion that a histological proof should be obtained if possible. There may be exceptions sometimes depending on local conditions. In endemic areas with one specific *TTR* mutation, high penetrance and a relative monomorphous presentation of ATTR amyloidosis, diagnosis may sometimes be safe without a histological proof. This may be the case in some areas of Portugal. In most parts of the world, however, cases of systemic amyloidosis are usually scattered and a histological proof should be recommended.

The site of biopsy may be discussed. We perform subcutaneous adipose tissue biopsies (surgical or by punch technique) with good results. Fine needle biopsy is not recommended since with this technique very little connective tissue is obtained, which is the site of ATTR amyloid containing TTR fragments (2). In other centers, labial salivary gland biopsies are preferred. Gastrointestinal tract is another option. A substantial advantage with biopsy techniques is that the biochemical type can be determined directly.

A question to discuss is whether indirect techniques such as DPD scintigraphy, SAP scintigraphy or PET with various ligands are safe enough for a definite amyloid diagnosis. Also alternatives to Congo red are under development but not yet validated enough for clinical use.
